# Mechanics of Pollen Tube Elongation: A Perspective

**DOI:** 10.3389/fpls.2020.589712

**Published:** 2020-10-20

**Authors:** Prakash Babu Adhikari, Xiaoyan Liu, Ryushiro D. Kasahara

**Affiliations:** ^1^School of Life Sciences, Fujian Agriculture and Forestry University, Fuzhou, China; ^2^Horticultural Plant Biology and Metabolomics Center (HBMC), Fujian Agriculture and Forestry University, Fuzhou, China

**Keywords:** mechanosensors, tip elongation, durotaxis, kiss–and–run, callus plug, pollen tube

## Abstract

Pollen tube (PT) serves as a vehicle that delivers male gametes (sperm cells) to a female gametophyte during double fertilization, which eventually leads to the seed formation. It is one of the fastest elongating structures in plants. Normally, PTs traverse through the extracellular matrix at the transmitting tract after penetrating the stigma. While the endeavor may appear simple, the molecular processes and mechanics of the PT elongation is yet to be fully resolved. Although it is the most studied “tip–growing” structure in plants, several features of the structure (e.g., Membrane dynamics, growth behavior, mechanosensing etc.) are only partially understood. In many aspects, PTs are still considered as a tissue rather than a “unique cell.” In this review, we have attempted to discuss mainly on the mechanics behind PT–elongation and briefly on the molecular players involved in the process. Four aspects of PTs are particularly discussed: the PT as a cell, its membrane dynamics, mechanics of its elongation, and the potential mechanosensors involved in its elongation based on relevant findings in both plant and non-plant models.

## Introduction

The pollen tube (PT) is a unique and specialized structure in plants. Its sole purpose is to deliver sperm cells to the female gametophyte for double fertilization. Essentially, it is a thread–like structure spanning from the pollen shell toward the tip. Generally, angiosperm PTs penetrate the cuticle and cell wall of the stigmatic papillae and then pass through the nutrient–rich extracellular matrix or periplasmic space of papillae and transmitting tract (TT) toward the female gametophyte (Lora et al., 2010; Ndinyanka Fabrice et al., 2017). Its distant relatives (e.g., cycads and Ginkgoales members), do not produce pollens. Instead, their sperms show zooidogamy by which they swim in the film of water toward the female gametophyte (Friedman, 1990). In most of the other gymnosperms, the PT grows significantly shorter (often grows on the surface of female gametophyte) and at a much slower rate (<20 μm/h), as compared with that in angiosperms (<3.3 μm/s in maize) (House and Nelson, 1958; Williams, 2008). PT–elongation significantly varies among angiosperms as well. Although it is generally unbranched, the PTs of the members of some taxa (e.g., Cucurbitaceae) often branch after entering the ovule (before fertilization) and may exhibit a haustorial nature (Johri, 1992). Even within the same species, PT elongation rate may vary between *in vivo* and *in vitro* conditions (<3.3 and <0.9 μm/s, respectively, in maize) (House and Nelson, 1958; Kliwer and Dresselhaus, 2010). Additionally, the elongation rate oscillates within a single PT which, in lily, may range from 0.1 to 0.5 μm/s (*in vitro*) in a period of 15–50 s (Cárdenas et al., 2008).

*In vitro* studies suggest that PTs should reach 100–200 μm vicinity to perceive female gametophyte cues (Dresselhaus et al., 2011), whereas *in vivo* studies have shown that female gametophyte–derived cues can reach much further and play a role in PT–redirection from the TT to the funiculus (Meng et al., 2019; Zhong et al., 2019). According to a recent study, the PTs of several species exhibit durotactic growth (faster elongation at stiffer matrices), whereas others do not (Malik et al., 2020; Reimann et al., 2020). Even with such diversity, all PTs share a common purpose, that is, to safely deliver sperm cells to the female gametophyte for double fertilization. Sperm cells remain as a passive cargo during its whole journey (Zhang et al., 2017) and PT elongation and redirection are driven solely by the cellular machineries at the PT cytoplasm which play a crucial role during double fertilization as well (Kawashima et al., 2014; Kasahara et al., 2016, 2017). Although there have been several studies on PT elongation and molecular/chemical players involved in the process, the whole picture is far from complete. Moreover, mechanics of its elongation is poorly understood. In this review, we discuss several persisting and unfolding issues regarding the nature of PT and mechanics of its response at its growth environment based on the findings in relevant fields.

## Pollen Tube: A Growing or a Moving Cell?

**FIGURE 1:**
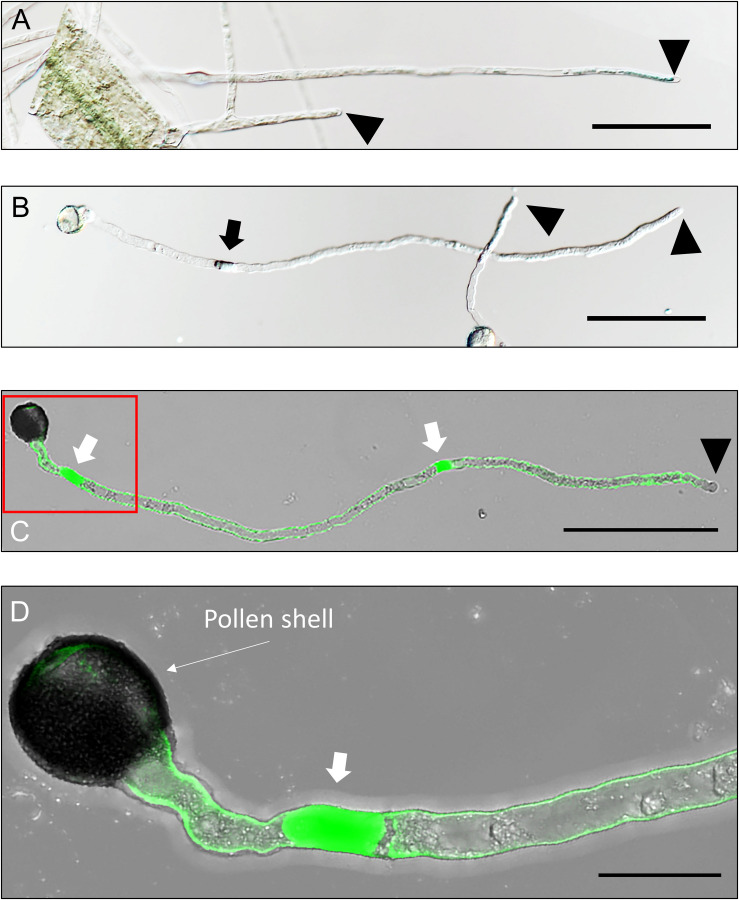
*Arabidopsis* root hairs and pollen tubes (PTs). Root hairs and PTs are morphologically very similar (**A,B**, respectively). However, cytoplasm of a growing, single celled root hair always remains connected to its mother cell **(A)** while elongating PTs have callose deposited at regular interval thereby disconnecting their front region from the spent growth **(B–D)**. A aniline blue treated PT shows its inner callose-layer of its cell wall except in its extreme tip **(C)**. The red-demarcated region in **(C)** has been magnified in **(D)**. Arrowheads point to the apex of root hairs or PTs and arrows point to the callose plugs in PTs. Scale bars: **(A–C)**: 100 μm; **(D)**: 20 μm.

In vascular plants (among all eukaryotes), pollen and PT are the only such structures that harbor cells (generative cell and sperm cells, respectively) within a cell (vegetative cell) when the mitochondria and plastids are taken as cell components (as they are). Apparently, the PT “grows” longitudinally, toward the female gametophyte in style, which, in many aspects, closely resembles any other tip-growing structures. Such structures include hyphae in fungi, rhizoids in algae and several spore–producing plants (moss and fern), protonemata in spore producing plants, root hairs in vascular plants; and neuronal axons in animals. All tip–growing structures exhibit tip–focused active metabolism and oscillatory polar growth (Albus et al., 2013; Rounds and Bezanilla, 2013; Takeshita, 2018); however, they vary from each other based on their purpose. One major difference between PTs (and protonemata) and other tip-growing structures is that unlike others wherein the cytoplasm retracts and is salvaged by the host tissue unless the cytoplasm is too damaged during programmed cell death (Logi et al., 1998; Hagg, 1999; Kacprzyk and McCabe, 2015), PTs lack such feature. An elongating angiosperm PT is a one–way flow, which appears more like a moving cell than a growing tissue as the back–flow of its content is blocked by the callose deposition at regular interval (Figure 1).

**FIGURE 2:**
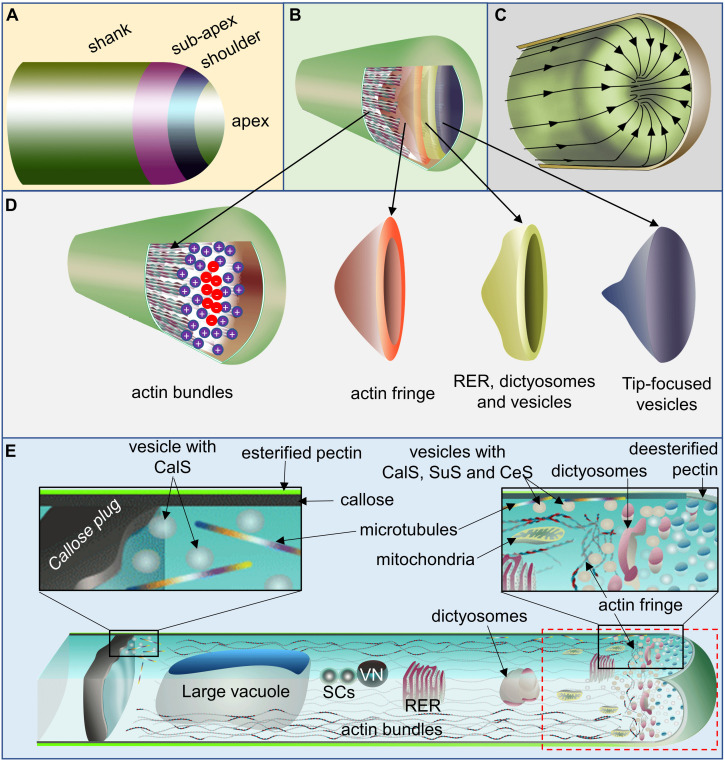
Schematic representation of the pollen tube (PT) front region. **(A)** PT with its tentative four zones. **(B,D)** PT with its portion of its cell wall cut opened to show actin bundles at alternate directions [barbed end (+) facing apex at the cortex and pointed end (–) facing apex at the core], exclusive vesicle population at the extreme apex encircled by organellar population along vesicles and by the actin fringe. **(C)** PT with its cell wall cut opened to show the cytoplasmic stream inside which flows toward apex at the cortex and reverses its direction at the subapical zone and flows back at its core giving a reverse fountain-like appearance. **(E)** A representative PT with its upper and front section of the shank (from viewer’s perspective) cut opened to show its cytosolic components. It comprises callose plug distally formation of which depend on microtubule-assisted incorporation of CalS at the site close (and distal) to the large vacuole. Two sperm cells linked to the VN move in front of the large vacuole. Cortex comprises of actin bundles with its barbed end oriented toward PT-apex while that of the actin at the core is oriented toward large vacuole. Actomyosin-assisted organellar flow occurs at this region in flow motion illustrated at **(C)**. The sub-apical region harbors shorter F-actin fringe and organellar population of mitochondria, dictyosomes, rough endoplasmic reticulum (RER), vesicles etc. while extreme apex comprises of vesicles exclusively in conical shape as shown in **(B,D)**. The positions of cytosolic components are not to the true scale. CalS, callose synthase; SuS, sucrose synthase; CeS, cellulose synthase; SCs, sperm cells; VN, vegetative nucleus.

While discussing the function of actin earlier, Steer (1990) indicated that the PT tip retains many features of the primitive amoeboid motion. Later observation of the PT tip elongation, even in the absence of its spent growth and host pollen in lily, further strengthened such postulation (Jauh and Lord, 1995). Structurally, the PT can be divided into four distinct zones, namely, apex, shoulder, sub–apex, and shank (Figure 2A). Multiple direct and indirect observational studies have shown that a typical elongating–PT comprises of actin bundles at its cortex (barbed–end facing PT–apex) and center (pointed–end facing PT–apex) in its shank region up to the subapical zone (Figures 2B,D). Its apex is populated with vesicles in a conical shape, which is encircled with the organellar population including vesicles, which itself is encircled by an actin fringe (Figures 2B,D,E) (Bove et al., 2008; Stephan, 2017). The organelles move forward at the cortex of the PT–shank and rearward at its center, forming a reverse fountain–like appearance (Figure 2C), although larger organelles reverse their path near sub–apical zone, mitochondria and dictyosomes and rough endoplasmic reticulum reach up to the base of PT–tip dome. Additionally PT comprises a large vacuole at its distal end, in front of which reside two sperm cells linked to the vegetative nucleus (Figure 2E) (Derksen et al., 1995, 2002; Bove et al., 2008; Cai and Cresti, 2008; Zhu et al., 2014a; Ndinyanka Fabrice et al., 2017; Yanagisawa et al., 2017).

### Cell Wall: Unique Coat for a Unique Structure

Studies in tobacco showed that PTs constitute 75%–88% callose, 6%–15% arabinan and just 6–19% cellulose (Schlüpmann et al., 1993). Of the two cell wall layers, the outer pectin layer remains esterified at the PT–tip which gets de–esterified distally starting from the sub–apical zone (Figure 2E). The inner callose layer is deposited at the tip–shank junction (5–10 μm distance from the tip in *Arabidopsis*) (Cai et al., 2011; Chebli et al., 2012; Hill et al., 2012). Callose is crucial for cushioning the elongating PT against tensile and compression stresses (Parre and Geitmann, 2005). Unlike in PT, the primary cell wall of root hairs comprises randomly oriented cellulose microfibrils (soft) at its tip, and the tough secondary cell wall (comprising cellulose fibrils in a parallel orientation to the growth axis) at the shank (Hirano et al., 2018).

**FIGURE 3:**
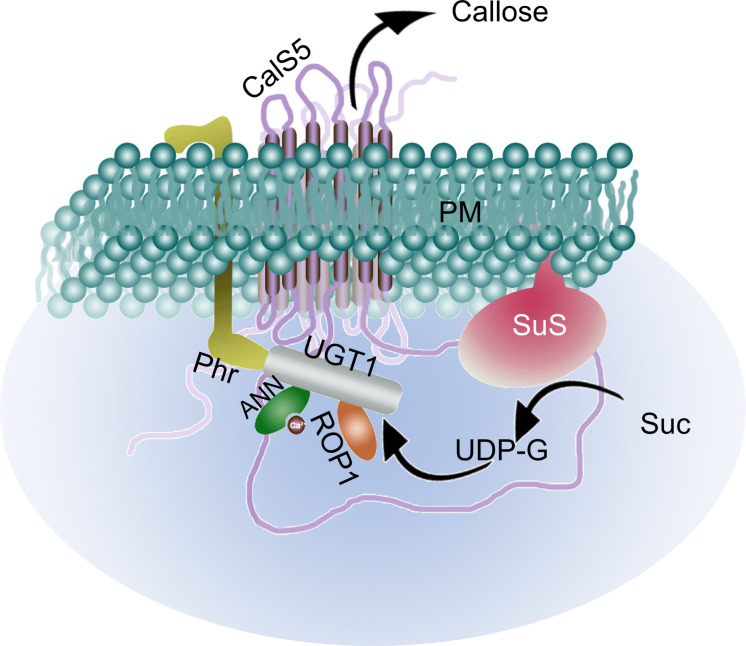
Putative callose synthase complex model with known PT-specific and other components. UGT1 interacts with CalS5 and act as its subunit. Membrane bound Phr interacts with UGT1 as well. The complex is activated by Rop1 after which the UDP-G synthesized by SuS is converted to callose and released out of the cell. The Ca^2+^ bound ANN inhibit the process by interacting with UGT1 and hence the callose synthesis process fluctuates with the Ca^2+^ oscillation inside the cell. PM, plasma membrane; CalS5, CALLOSE SYNTHASE5; UGT1, UDP-glucosyltransferase1; Phr, phragmoplastin; Rop1, Rho-like GTpase1; ANN, annexin; UDP-G, UDP-glucose; SuS, sucrose synthase; Suc, sucrose.

Studies have shown that the cellulose synthase complex harboring multiple cellulose synthase catalytic subunits, callose synthase complex, and sucrose synthase are carried by the dictyosomes along the actin bundles up to the sub–apical zone, which moves along the actin filaments of the actin fringe, and is later incorporated into the plasma membrane near the tip (Cai et al., 2011). The callose deposition in the elongating PTs starts beyond the sub-apical region, approximately 20 μm from the tip in tobacco (Derksen et al., 1995). The callose synthase complex of PTs consists of CALLOSE SYNTHASE5 (CalS5) that harbors two clusters of transmembrane domains flanking a long hydrophilic domain (Abercrombie et al., 2011). According to the widely accepted model proposed by Verma and Hong (2001), UGT1 acts as a subunit of callose synthase complex, and interacts with the membrane bound phragmoplastin (Phr), a cell plate–associated protein. It additionally interacts with Rho–like GTpase1 (ROP1) which regulates the callose synthesis process (Hong et al., 2001). After the membrane bound sucrose synthase synthesizes UDP-glucose, the ROP1-activated callose synthase complex converts it to callose and releases across the plasma membrane (Figure 3). An *in vivo* study (cell culture) in *Arabidopsis* showed that Ca^2+^ plays a quintessential role for the callose synthase activity which is further stimulated by Mg^2+^ (Aidemark et al., 2009). The study in cotton fiber additionally showed potential binding of annexins (ANN) with the CalS in a Ca^2+^–dependent manner thereby inhibiting its function (Figure 3) (Andrawis et al., 1993). A pollen and PT–specific ANN, ANN5 has been reported in *Arabidopsis*, which is known to involve in the acto–myosin endomembrane trafficking (in a Ca^2+^ dependent manner) (Zhu et al., 2014a,b). Although it is most likely, the potential involvement of ANN5 or any other PT–specific ANN in callose synthesis has not been reported yet. A review by Dehors et al. (2019) discusses more on the cell wall components and evolution in tip-growing structures.

### Callus Plug: Partition-Structure Without Cell Division

A unique property of PT is its callose plug at regular intervals, which blocks the cytoplasmic connection to the distal PT region, with the newest plug close to the large vacuole (Figures 1C,D, 2E). Such feature may help the PT maintain its “cellular” feature while focusing its energy and machineries solely on the forward elongation of the front region. This is the reason why PT remains one of the fastest growing structure in plants (0.1–0.5 μm/s) (Hepler et al., 2013) unlike which root hairs grow at the rate of just 10–40 nm/s (Galway et al., 1997; Wymer et al., 1997).

Callose deposition at the inner cell wall layer keeps thickening toward the distal end and forms a plug at 40–100 μm distal to the tip (Figures 1C, 2E) (Lennon and Lord, 2000; Derksen et al., 2002; Chebli et al., 2012). The plug prevents the backflow of the PT contents thereby maintaining its turgor pressure and integrity (Li et al., 1997; Parre and Geitmann, 2005). Callose plugs are evolutionarily developed only in angiosperms as the PTs of gymnosperms lack such depositions (Williams, 2008). The interval and the topmost callose plugging sites may vary among different species and even among different ecotypes of the same species (Laitiainen et al., 2002; Mogami et al., 2006; Cai et al., 2011; Qin et al., 2012). As observed in tobacco, the continuous movement of the vegetative nucleus and generative cells toward the tip in the elongating PT is affected when treated with oryzalin, a microtubule polymerization–inhibiting dinitroaniline herbicide with high affinity to plant tubulin monomers (Morejohn et al., 1987; Laitiainen et al., 2002). The intracellular location of microtubules is reported to be a determinant factor for the site of plug formation in PTs. They are known to incorporate the callose synthase complex in the plasma membrane around the site of plug formation distal to the large vacuole, as observed in tobacco PT (Cai et al., 2011). To date, what triggers the machinery to initiate plug formation at a certain interval is unclear. Observing the nature of PT elongation, one possible factor would be the internal turgor pressure, provided that it oscillates with the PT elongation. However, studies show that turgor pressure is not correlated with the elongation rate of the PT, although the pressure may slightly vary within it (Benkert et al., 1997; Kroeger et al., 2011). Furthermore, changes in PT length per oscillatory cycle of its elongation is much shorter than the distance between two callose plugs (Benkert et al., 1997; Kroeger et al., 2011; Qin et al., 2012), indicating negligible, if not, null effect of the turgor pressure on the plug formation. A related study has further reported constant turgor pressure in growing lily PTs (Hill et al., 2012). In such case, the most probable reason for the initiation of callose plug formation may be linked with the concentration gradient of the enzymes that are directly/indirectly involved in microtubule mobilization and/or callose synthase complex incorporation in the plasma membrane at the plug formation site. This unknown enzyme may be controlled by a negative feedback loop in such a way that its function/expression would be impaired at its higher concentrations.

## Membrane Dynamics: Exocytosis and Endocytosis

### Processes of Membrane Recycling

As previously mentioned, PTs have a relatively low cellulose content, which is recycled within the short front region (Mogami et al., 2006). The recycling involves new membrane incorporation to and membrane retrieval from the plasma membrane, mainly *via* exocytosis and endocytosis (respectively). A strongly held belief for PT membrane recycling (being debated lately) assumes that endocytosis is largely restricted at the sub–apical region, whereas exocytosis is restricted at the apex. However, for a fast–elongating structure, concentrating most of its exocytotic activities at the apex (that would give drag to its elongation by spewing vesicular contents out) and endocytotic activities at the sub–apical region seemed like an ill–designed natural structure.

**FIGURE 4:**
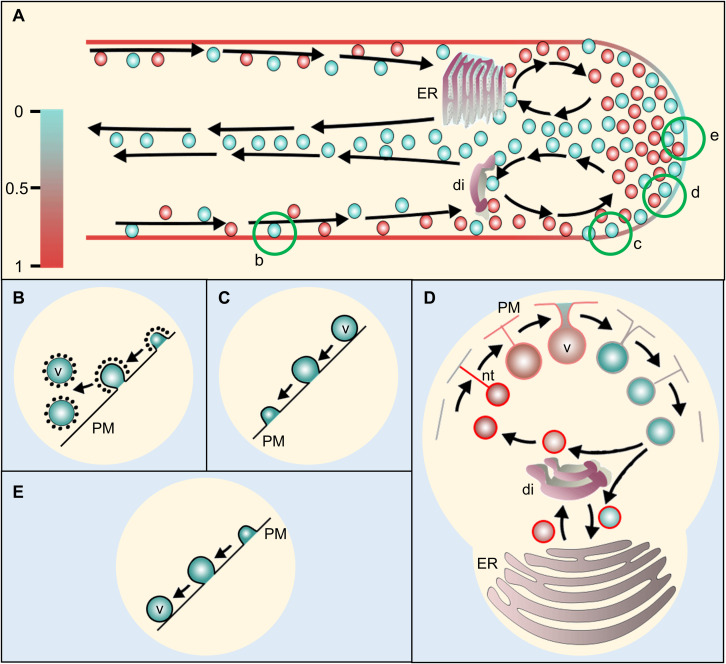
Proposed model of membrane recycling in pollen tube (PT). **(A)** A schematic representation of a pollen tube with its vesicle dynamics. Arrows show the path of the vesicle movement. The scale at the left represents color code for relative membrane tension at PT plasma membrane and vesicles for **(A,D)** only. The vesicles arriving from the cytoplasmic stream at PT-cortex move toward apex. Those returning from the apex may return back or fuse with dictyosomes/RER (rough endoplasmic reticulum) or flow distally along the PT core. The dictyosomes and RER may bud-off smaller vesicles, which then move toward the apex along the flow. The apical and sub-apical regions may serve for both exocytosis and endocytosis with their different modes at certain segment depending on the PT membrane tension. Conventional clathrin-mediated endocytosis (CME) **(B)** takes place at distal region in PT shank in which plasma membrane is actively retrieved (Bove et al., 2008; Zonia and Munnik, 2008, 2009). Fully distended exocytosis **(C)** occurs at the subapical to lower shoulder region which increases PT membrane area and excess membrane is pushed toward the apex. Further at the upper shoulder region, kiss-and-run mode of exocytosis/endocytosis **(D)** may occur due to the decreased ratio of PT-membrane to vesicle-membrane tension. During the process, the vesicle never reaches to the plasma membrane but gets connected with a lipid nanotube, through which, lipid flows toward the vesicle thereby equilibrating the vesicle membrane tension with the PT plasma membrane (Mellander et al., 2014) while releasing some of its content and picking up some extracellular matrices during the process. When the tension of vesicle membrane equilibrates with that of the PT-tip, the nanotube connection breaks and the free vesicle moves inward at PT core **(A)** toward dictyosomes/RER. The budding vesicles from dictyosomes may enter the process all over again. At the extreme apex, smooth endocytosis **(E)** may occur through which, membrane is directly retrieved without CME while engulfing ECM during the process (Bove et al., 2008). ER, endoplasmic reticulum; di, dictyosomes; v, vesicle; PM, plasma membrane; nt, nanotube.

Interestingly, some indirect observational studies suggested that endocytosis is possibly tip–focused (in addition to the lateral endocytosis at the PT shank distally; Figures 4A,B), and exocytosis is probably restricted at the sub–apical and/or shoulder region (Figures 2A, 4A–E) (Bove et al., 2008; Zonia and Munnik, 2008, 2009). In a tobacco PT study, Zonia and Munnik (2008) used total internal reflection fluorescence microscopy (TIRFM) which selectively illuminates and excites the fluorophores of the specimen *via* evanescent waves (hence it is also called evanescent wave microscopy) (Axelrod, 1981). They tracked the newly endocytosed vesicles after treating the elongating tobacco PTs with a non-toxic lipophilic FM dye (FM 1–43, green) for 2 h followed by the treatment with another FM dye with a different emission wavelength (FM 4–64, red) and observation at 21 s intervals for up to 20 min. They observed that the mixed fluorescence was consistently detected in the apical vesicle–population, whereas full membrane distention was observed only on the sub-apical zone. From such observations, the authors argued that PT–apex is the exclusive site for endocytosis and membrane retrieval, whereas exocytosis is restricted in the zone adjacent to the apical dome. Furthermore, authors suggested that the exocytosed membrane pushes toward both directions (apically as well as distally) thereby providing enough membrane for PT–shank elongation and apical membrane internalizing (Zonia and Munnik, 2008, 2009).

An almost simultaneous study by Bove et al. (2008) used different approach and had a slightly different conclusion about the vesicular trafficking at PT–apex (lily). The authors used spatiotemporal image correlation spectroscopy (STICS) and fluorescence recovery after photobleaching (FRAP) to retrieve the vectors of vesicle movement in the PTs dyed with FM1–43. They observed the accumulation of vesicles arriving from cortical cytoplasmic stream at the shoulder of the apex, but found that the extreme apex never fluoresced with full intensity. Based on their observations, the authors argued that the vesicles may requires “more than one passage” to reach to the apex and fuse to the plasma membrane and proposed that “kiss–and–run”–mode of exocytosis may explain such observation, wherein the vesicles undergo transient fusion without fully integrating to the plasma membrane while releasing some vesicular content in the process. Additionally, they proposed smooth endocytosis at the extreme apex (Figures 4A,E) (Bove et al., 2008).

The observations and postulations made by Zonia and Munnik (2008), as well as Bove et al. (2008) also explain the potential disparity observed by the earlier studies (with the assumption that all tip–localized vesicles are fully fused with the plasma membrane during exocytosis), which reported that PTs add more membrane to the plasma membrane than what is required for their elongation and endocytosis (Picton and Steer, 1983; Steer, 1988; Derksen et al., 1995; Ketelaar et al., 2008). In their earlier tobacco PT electron micrographs observational study, Derksen et al. (1995) found that the fusion rate of the tip–localized (smooth morphology) putative secretory vesicles in PT accounted for 430 μm^2^/min, whereas that of putatively endocytosed vesicles (rough morphology, receptor coated) behind the tip accounted for 225 μm^2^/min. However, the expansion rate of the PT (elongation rate: 2–3 μm/min; diameter: ∼8 μm) was just 50 μm^2^/min and left an unaccounted excess membrane of 155 μm^2^/min. Interestingly, they observed that PTs encompass smaller vesicles (in large population) at their apex. However, the coated vesicles close to plasma membrane was relatively larger than those close to the dictyosomes (Derksen et al., 1995).

Relatively recent study by Prado et al. (2014) on olive showed that PTs directly secrete nanovesicles of 28–60 nm (referred to as pollenosomes). Secretion of such membrane bound vesicle may account for some proportion of the disparity in membrane turnover observed earlier. However, for a fast elongating structure, like PT, removal of about one–third of its newly added membrane is still questionable.

Disparity in membrane turnover had also been reported for growing root hairs, which led early researchers to contemplate that the excess membrane materials may be actively destroyed by the cellular machineries (Steer, 1988). However, the study by Ketelaar et al. (2008) showed that PTs can sustain elongation for the estimated time (33 s) even after the new exocytotic vesicle formation is blocked with cytochalasin D (in *Arabidopsis*). Their observation indicated that the elongating PT (and possibly the growing root hair as well) is less likely to destroy (or secrete) its plasma membrane in significant proportion. Furthermore, the authors also suggested that the vesicles may undergo partial (kiss-and-run) or full distention with the plasma membrane, depending on the membrane requirement and internal vesicle population.

Although multicellular, the disparity in coleoptile cell wall expansion and vesicle quantity had led to the discovery of the kiss-and-run mode of exocytosis in maize earlier (Weise et al., 2000). In their study, the authors measured membrane capacitance (*C*_m_) using patch–clamp techniques. The irreversible increase in *C*_m_ was observed as a result of vesicle fusion (taking >2 s) while the reversible one was taken as the result of the kiss-and-run type of transient vesicle fusion with the plasma membrane (completed within 100 ms) (Weise et al., 2000). Additional studies have shown that kiss-and-run mode can equally contribute to the endocytosis as well (Kavalali, 2009; Wen et al., 2017).

One recent study reported that pollen germination requires autophagy–mediated compartmental cytoplasmic deletion in tobacco (Zhao et al., 2020). Whether it acts as a part of membrane recycling machinery in elongating PT is yet unknown. Post–germination knockdown (or knock-out) studies of the responsible genes (*ATG2*, *5*, and *7*) may clarify it in the future. The reviews by Grebnev et al. (2017) and Guo and Yang (2020) focuses specifically on the PT membrane dynamics in bit more detail.

### Mechanics of the Unconventional Mode of Membrane Recycling (Kiss-and-Run Mode)

Discussions on the mechanisms of fully distended exocytosis and endocytosis is relatively more prevalent (Onelli and Moscatelli, 2013; Grebnev et al., 2017; Guo and Yang, 2020) than that on the kiss-and-run mode of exocytosis/endocytosis. Thus, we have attempted to discuss potential causes and mechanisms of kiss-and-run mode of exocytosis/endocytosis in this section.

An observational membrane fusion study on artificial cells (protein–free liposome system) earlier led to the confirmation of the two modes of exocytosis: first, full distention, wherein, smaller daughter vesicles were completely fused to the membrane of the mother vesicle (harboring the former in the inside) after the lipid nanotube formed between them extinguished leading the vesicle to grow larger under pressure; and second, the partial distention, wherein, the daughter vesicle never reached to the cell membrane, but formed a lipid nanotube between them which transiently enlarged allowing some contents of the vesicles to be released, followed by narrowing the nanotube again (comparable to Figures 4A,D) (Mellander et al., 2014). The latter form was similar to the kiss-and-run mode of exocytosis, but with extended time and pore opening. Furthermore, they observed that the size of the vesicles that underwent partial distention was relatively smaller than those that underwent full distention, and that the relative membrane tension (between the daughter and mother vesicles) plays a determining role on which mode of exocytosis would follow. They proposed that during partial distention, lipid flows from the mother vesicle to the growing daughter vesicle when the latter has higher membrane tension, which stops at the equilibrium. Further increase in the daughter vesicle’s size leads to the contraction of the mother cell, thereby increasing the pore (nanotube) size and reducing pressure in the daughter vesicle, which subsequently reduces the pore opening back to the nanotube before it completely releases its content (Mellander et al., 2014), and The process can be very different at *in vivo* conditions, in which many cellular and molecular players actively play role in membrane dynamics.

Studies in PT show that its tip has the lowest plasma membrane tension and stiffness which steeply increases toward the shank (Figure 4A) (Hepler et al., 2013; Vogler et al., 2013), suggesting that the vesicles near the PT apex possibly undergo kiss-and-run mode of exocytosis/endocytosis instead of full distention. Although the lowest stiffness has often been described as the “result” of apical exocytosis (Hepler et al., 2013), it being the “cause” for potential apical endocytosis has not been contemplated yet. It is probable that the dictyosomes and rough endoplasmic reticulum, at the organellar cone, bud-off smaller vesicles which would undergo kiss-and-run mode of exocytosis/endocytosis at the near PT-shoulder and apex, flows back and fuse to them or move along the core cytoplasmic stream (Figures 2B–E, 4A). Could the observation made by Derksen et al. (1995) of the vesicles close to dictyosomes being smaller than those close to plasma membrane in PT be due to such occurrences? It demands further evidences to support such assumption at the moment.

## Pollen Tube Elongation: Correlation to Single Cell Migration

PT elongation is closely linked to the membrane recycling we discussed earlier. Its directional elongation relies on the internalization of external cues, triggering series of molecular chain reaction, that leads to cytoskeletal rearrangement and change in angle of vesicle–population positioning at the apex (Bove et al., 2008; Bou Daher and Geitmann, 2011). In this section, we discuss on how PT translates its interaction with external surface to its elongation rate.

### Durotaxis: Pollen Tube Steals a Cellular Move

A recent PT–elongation study by Reimann et al. (2020) showed that the PTs of the plants with solid or semi–solid TTs (sPTs) show increased elongation rates in stiffer matrices. The PTs of the plants with hollow TTs (hPTs), however, showed opposite behavior. Interestingly, the force exerted by the elongating sPTs was recorded far lower (<11 μN for tobacco PT) than that of their hPT counterparts (<36 μN for lily PT) (Reimann et al., 2020). However, the elongation of both types of PTs was halted at excessively high matrix stiffness level (12% agarose). Such behavior of exhibiting increased movement speed in stiffer materials is referred to as “durotropic” movement (Sunyer and Trepat, 2020) and has been observed by many other researchers (almost all in non-plant models) for single cell migration, after the first study was reported by Lo et al. (2000) on fibroblasts. However, not all cells show a positive response to the stiffer matrices in terms of their movement speed. Recent mathematical modeling for a single-cell migration on an elastic matrix suggests that durotactic motion is determined by the ratio of the stiffness gradient to the absolute stiffness of the growth matrix (which is elastic and deformed under the force exerted by the migrating cell), and lifetime of the focal adhesions (membrane site which adheres to the matrix) depends on the force exerted by the migrating cell (Malik et al., 2020). While no such focal adhesions has been reported in PTs yet, their durotropic nature of elongation suggests for similar, if not same structure or phenomenon in them.

Contradictory to the observation made by Reimann et al. (2020), an earlier report by Haduch-Sendecka et al. (2014) showed an opposite responses of tobacco PT (sPT) at stiffer agar, which could be due to different properties of the agarose used in these two studies. Since most of the PT–related microscopic observations and analytic studies have been conducted on the *in vitro* cultured PTs, they may not exhibit true behavior of their *in vivo* counterparts. The nutrient-rich extracellular matrix does not only provide a path for PT elongation but also actively supports and augments the process (Lord, 2000, 2003; Qin et al., 2011). PTs internalize the extracellular matrix as they elongate through the TT (Cheung and Wu, 2006; Kim et al., 2006). From the stigma–specific self–compatibility related genes to TT–specific *AGP* and *TTS* (Cheung et al., 1995; Wu et al., 1995), and female gametophyte–specific *MYB98* (Kasahara et al., 2005), *LURE* (Okuda et al., 2009; Takeuchi and Higashiyama, 2016), *XIUQIU* (Zhong et al., 2019), etc. all play crucial roles in a precisely orchestrated fashion to drive PT elongation and direct it toward female gametophyte.

The different responses of sPTs and hPTs to the matrix stiffness, observed by Reimann et al. (2020), may have been evolutionarily developed, as the former have to pass through the extracellular matrix at the solid style and the latter have to stick to the cells (beneath the cuticle) and elongate forward at the inner wall of the hollow style.

### Modes of Cellular Movement: Reference From Non-plant Models

The single–cell movement characteristics are not discussed that much in plants, since cells are often encapsulated in a hard cell wall. However, several observations on single cell movement in animal or microbe models are interestingly similar to the PT elongation. A micropipette aspiration study on *Entamoeba histolytica* (a unicellular amoeboid parasite responsible for amebiasis in animals) earlier showed how capillary suction pressure and wall friction play positive roles in the parasite’s forward motion by blebbing, a process of cellular movement characterized by membrane protrusion and contraction (Brugués et al., 2010). During blebbing, membrane protrusion (bleb) is initiated either through the localized detachment of plasma membrane from the acto–myosin cortex or the local cortical rupture. Cellular turgor, without any supporting cortex under the membrane at the protrusion, leads the bleb to expand while further detaching away from the cortex at its base. After a new actin cortex is formed under the bleb membrane and myosin is recruited, the bleb retracts. By repeating these steps, cells exhibit a blebbing movement (Charras and Paluch, 2008). Previous study by Brugués et al. (2010) suggested that the acto–myosin cortical thickness determines the movement pattern of the blebs. They postulated that cells exhibit oscillatory blebbing when the value for its critical cortex (acto–myosin) thickness required for the membrane unbinding exceeds to what is required for the membrane retraction (whereas that required for the stationary membrane remains the highest) (Brugués et al., 2010).

The model used in the study was very different from PT, and the oscillatory blebbing movement reported in *E. histolytica* is only roughly comparable to the PT (tip) movement/elongation. Although pressure/tension-induced cortex rupture is not observed in PT, its tip essentially lacks the cortical support. PT tips cultured in hypoosmotic/hypotonic solution exhibit typical club–head structure (Zonia and Munnik, 2008) indicating the acto-myosin cortex supported shank with methyl–de-esterified wall and cortex-free tip without cytoskeletal support. Moreover, actin dynamics (de–/polymerization) at its sub-apical zone is known to play a key role in its elongation. Thick actin bundles and fine actin filaments are characteristic components of the shank and apex/sub–apex region (respectively) of a normally elongating PT (Kost et al., 1999).

In most angiosperms, the stigma–style interface is embedded with densely packed cells exposing a reduced secretory surface for growing PTs. Hence, they need to elongate invasively to pass through the interface (Lora et al., 2010; Ndinyanka Fabrice et al., 2017). A recent study on the elongation of *Torenia fournieri* PTs, *Arabidopsis* root hairs, and *Physcomitrella patens* protonemata (each with the diameters of 8, 8, and 18–20 μm) on microchannels showed that the PT–tip is the weakest of them and often ruptures or gets stuck in the middle of the channel when elongating through too long pores with the gap of 1 μm (Yanagisawa et al., 2017). Interestingly, all of the tissues showed deformations while passing through the narrow gaps. PTs may undergo similar constraint at the stigma–style junction and narrow intracellular spaces at TT. The elongating PTs that are adjacent to one another, often share a common cell wall (Lennon et al., 1998; Lord, 2000; Ndinyanka Fabrice et al., 2017). How such surroundings affect PT-elongation physically and their potential involvement in its durotactic nature are yet to be elucidated.

Additionally, unlike sPTs, hPTs (lily) grown *in vivo* were often reported to have star–shaped actin foci at the site of PT shank where adhesion event occur with adjacent PTs or TT cells as PTs elongate under the cuticle along the inner wall of the hollow TT (Jauh and Lord, 1995). It is interesting to think that these foci are the adhesion foci and are key to the movement/elongation of hPTs by “adhere–and–push–itself–forward” mechanism. Again, this type of movement has been frequently discussed and observed in animal cells, and termed as “lamellipodial movement.” The possible role of this type of movement in PT and root hair growth has earlier been contemplated though (van der Honing et al., 2007). During lamellipodial movement, the actin layer above the less dynamic actin bundles at the cortex gets polymerized at the front, thereby evoking its contraction until the actin network reaches the adhesion site where myosin II–cluster is formed, which plays a key role in bending the upper dynamic actin layer, leading to edge retraction and initiation of a new adhesion site (Giannone et al., 2007).

Observations in lily and tobacco showed that PTs harbor myosin II besides myosins IA, IB, and V, that were all suggested to move larger organelles in PTs (Miller et al., 1995). Myosin II is known to play role in the protrusion and contraction of cell and its attachment to the matrix or other cells during its movement in non-plant models (Sayyad et al., 2015). However, its potential role in PT adhesion has not been reported/discussed to date. Additionally, the highly dynamic actin filaments in PTs are localized at the sub–apical region, whereas its shank comprises actin bundles in an opposite orientation (pointed to barbed end at the cortex and barbed to pointed end at the center) (Figure 2D) (Lenartowska and Michalska, 2008). Furthermore, CAP1, a protein known to play a role in cell adhesion (Zhang et al., 2013), has been reportedly involved in apical actin polymerization in PTs (Jiang et al., 2019). Whether it plays role in PT–adhesion as reported in animal models (Zhang et al., 2013) is yet unknown.

Furthermore, the focal adhesion dynamics in the spreading cells is reportedly regulated by integrin ligands in animal models. A study showed that the cells plated on media nano-patterned with RGD (argenine–glycine–aspartate) nanoparticles at longer intervals (108 nm) spread slower as compared to their counterparts cultured at plate nanopatterned with RGD at shorter interval (58– nm) indicating the crucial importance of matrix, and available integrin binding sites for their migration (Cavalcanti-Adam et al., 2007). Studies have shown that growing PTs have integrin–like proteins localized at their tip near the plasma membrane, as observed at 5 and 10 μm in tobacco and lily, respectively (Sun et al., 2000). An interesting finding of the study was that tobacco PT–elongation was decreased *in vivo* in stigma [after it was pre–treated with RGDs or human placenta integrin vitronectin receptor (VnR) antibodies] by up to 36% and style (after the microinjection with RGDs or VnR antibodies) by up to 37% in a concentration-dependent manner while it remained unaffected *in vitro* indicating that the molecules disrupt the interaction between PT and the stigma/TT. However, PT–elongation remained uninhibited even at very high concentrations of RGDs or the antibodies (Sun et al., 2000). Another interesting observation of the study was that the tobacco PTs were immunostained with anti–β_1_ antibody, but not anti–β_3_, and anti–α_v_ integrin antibodies, whereas those of lily were immunostained with the latter two, but not with the anti–β_1_ antibody, indicating differences in their respective integrin-like proteins. Could the reduced elongation observed in the study be due to the lack of available active domains of integrin-like proteins which would otherwise interact with the stigma and TT-specific ligands to adhere? It is probable that the durotropic response of the hPTs observed by Reimann et al. (2020) would be affected by their treatment with RGDs/VnR *in vitro*. However, it may require further evidence to confirm such postulations.

Reports show that blebbing requires relatively lesser energy as compared to the lamellipodial movement (Charras and Paluch, 2008; Bovellan et al., 2010). Moreover, blebbing led movement is relatively faster than the lamellipodial movement (Charras and Paluch, 2008; Ikenouchi and Aoki, 2017). Additionally, cells (mesenchyma) are reported to exhibit faster movement/migration with low adhesion at confinement *via* blebbing (Liu et al., 2015). Such phenomenon of cellular movement appear to be a close parallel to the faster rate of sPTs elongation with lesser energy (as compared to hPTs) observed by Reimann et al. (2020). Moreover, PTs have inner layer of callose in their cell wall which cushions the forward flowing cytoplasmic contents by resisting the compression stress (Parre and Geitmann, 2005). It is possible that sPTs favor blebbing–like movement while hPTs favor lamellipodial movement. Future experimental evidences may shed more light on the aspect.

Animal-derived single cells often transit from one mode of movement to another during their migration (Bergert et al., 2012). Not all cells exhibiting lamellipodial (or blebbing) movement would necessarily show increased movement at stiffer matrices as the movement patterns largely depend on cellular machinery organization (at the focal adhesion) and the matrix stiffness (Malik et al., 2020; Sunyer and Trepat, 2020). Furthermore, studies showed that cells may exhibit lobopodial movement, an intermediate between lamellipodia, and blebbing movements, wherein cells migrate by forming tube–shaped lobopodia with protrusion comprising multiple blebs at the end/apex (Petrie et al., 2012; Petrie and Yamada, 2012), a movement characteristic closely resembling that of PT elongation.

## How Do Pollen Tubes Sense External Force/Tension?

Plants have mechanosensing mechanism that is not explored as much as that in their animal counterparts (Hamant and Haswell, 2017). One crucial plant cytoskeletal component responsive to mechanical stresses is the microtubule. It is known to direct cellulose microfibril deposition at the site of the plasma membrane under maximal stress by regulating the membrane incorporation of cellulose synthase complexes (Williamson, 1990; Kesten et al., 2017). As previously mentioned, the tip of the elongating PT has the lowest membrane tension that sharply increases toward the shank (Hepler et al., 2013). Furthermore, the apical methyl–esterified PT–wall starts de-esterification at the sub–apical zone, and microtubules–assisted incorporation of callose synthase complex, sucrose synthase, and cellulose synthase complex occurs at the site near the apex (Cai et al., 2011), the potential membrane site with highest membrane tension.

In animal model, RhoA–mDia1 signaling pathway is activated during the durotropic movement, leading to the formation of detyrosinated–microtubule network, thereby positively regulating adhesion site formation (Wang et al., 2017). Microtubules are proposed to be associated with the plasma membrane through p161 and with actin filaments through uncharacterized proteins (Cai and Cresti, 2008). Whether PT microtubules function in similar way remains to be elucidated.

**FIGURE 5:**
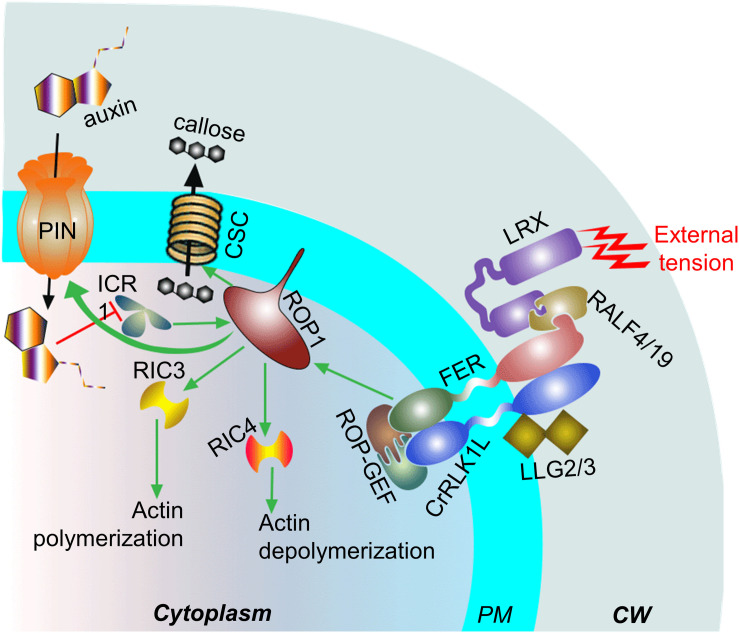
Potential players involved in mechanosensing at the tip of an elongating pollen tube (PT). The FER-complex (ANXUR1/2-BUPS1/2-LLG2/3) ‘senses’ external tension *via* cell wall (CW) localized LRX protein which interacts with FER-complex through RALF4/9. The plasma membrane (PM) localized FER promotes ROP1 activity, which in turn, regulates actin dynamics at the PT subapical zone through RIC3/4. The organization and reorganization of the actin may play crucial role in the PT-elongation as well as its adhesion to the external matrix. The membrane association of ROP1 is promoted by ICR1, interaction to which leads to an increase in auxin influx which, in turn, hinders ICR1 both transcriptionally and post-transcriptionally. ROP1 additionally activates the callose synthase complex (CSC). Green arrows represent positive effect, black arrows show flow of molecules, and red blunt-headed arrow represent negative effect.

Relatively recent evidences suggest that cell expansion is triggered after the plasma membrane-localized FERONIA (FER) “senses” cell-wall tension by interacting with the cell wall localized LRX extensin proteins and pectin (for more details see Doblin et al., 2014; Du and Jiao, 2020). Studies on PTs showed that LRX proteins interact with RALF4/19 that, in turn, interacts with the ANXUR1/2 (PT–specific FER homologs)–BUPS1/2 (CrRLK1L members)–LLG2/3 (LORELLEI-like-GPI-anchored proteins) complex at the plasma membrane (Figure 5). The ANXUR1/2–BUPS1/2–LLG2/3 complex is crucial for maintaining PT–tip integrity (see the review by Adhikari et al., 2020). A defect in *FER* leads to decreased levels of Rho GTPases (RAC/ROPs) and affects RAC/ROP–mediated, as well as NADPH oxidase–dependent, ROS production (Duan et al., 2010; Li et al., 2015). Rho GTPases are known to control protrusion of migrating cell and its adhesion to the matrix by modulating actin organization and reorganization in animal model (Sit and Manser, 2011; Warner et al., 2019). It is important to note that actin dynamics in extending PT-tip is also regulated by Rho GTPase member, ROP1 (ROP1P and ROP1At) by alternately activating Rho GTPase effectors, RIC3 and RIC4. The process is crucial for oscillatory PT elongation (Fu et al., 2001; Gu et al., 2005). As discussed earlier, ROP1 also regulates the process of callose synthesis at the tip-shank junction of an elongating PT, which is crucial for cushioning the structure against the external tension (Hong et al., 2001; Parre and Geitmann, 2005).

ROP1 additionally interacts with ICR1 (also known as RIP1), a Rho GTPase effector member, which promotes the plasma membrane association of ROP1 at the tip of elongating PT (tobacco) (Li et al., 2008). It is interesting to note that the interaction between ICR1 and ROP1 promotes the recruitment of PIN proteins (across membrane auxin influx pump) at the polar domain in plasma membrane. However, auxin affects the transcriptional and post-transcriptional regulation of ICR1 negatively (Wu et al., 2011). The enhanced biosynthesis and plasma membrane localization of the auxin efflux carrier PIN1 in response to mechanical strain has been reported in tomato shoot apices earlier (Nakayama et al., 2012). Yet another study in *Arabidopsis* roots suggested that FER regulates F–actin mediated PIN2 polar localization during gravitropic response (Dong et al., 2019). Auxin is known for its role in the directional growth of a tissue/cell. It inhibits endocytosis (Paciorek et al., 2005) and enhances exocytosis (including its own efflux) at the site of its higher gradient (Hager et al., 1991; Paciorek et al., 2005), which leads the tissue to bend/curve toward the side with lower auxin gradient (Went, 1974). A study in elongating tobacco PT showed that its tip is closely followed by the highest auxin gradient in transmitting tract (Chen and Zhao, 2008). The IAA-treated PTs (*Torenia fournieri*) elongating *in vitro* show their smooth and straight structure (as opposed to their characteristic wandering and kinked structure in absence of the hormone treatment) (Wu et al., 2008).

It is highly likely that the FER–related complex and auxin signaling are involved in the internalization and translation of external tensions/stresses in the elongating PT, triggering cellular responses to cope with it and ROP1 plays a pivotal role during the process. How they are translated and linked to each other as well as the potential involvement of microtubules in the process are yet to be fully elucidated.

## Conclusion and Perspective

It is interesting to view an angiosperm PT as a continuously moving elongated–cell at the tip of an “esterified pectin burrow,” wherein cytoplasm (and its machinery) is regularly partitioned from its distal region by callus deposition at certain intervals. Sperm cells serve little to no role in PT elongation and remain as a passive cargo during the whole process (Zhang et al., 2017) suggesting that PT is a putatively “elongated cell” with not three but essentially a single functional nucleus (vegetative nucleus).

Pollen tubes can be easily cultured *in vitro*; however, studies have shown that the features of *in vitro* PT are significantly different to that of its *in vivo* counterpart. *In vitro* PTs are basically the germinated PTs wandering around in search of a compatible stigma/papillae. An *in vitro* growth system that could closely mimic *in vivo* conditions would significantly improve the quality of PT study in the future. Recently discovered durotropic response of sPT shows its potential resemblance to single cell movement in non-plant models. How and if the PTs reorganize/change its intracellular components like actin, microtubules, integrin etc. during the process is yet to be determined. Future studies on ROP and its potential involvement on mechanosensing by linking FER-complex to auxin signaling and callose synthesis may also shed more light on the durotropic nature of the sPT elongation.

Pollen tube–apex reportedly comprises the smallest vesicles while those closer to membrane being relatively larger than those closer to dictyosomes (Derksen et al., 1995). Whether the kiss-and-run mode of exocytosis/endocytosis is the cause behind such occurrences, is yet to be fully elucidated. Potential approaches may include– use of membrane-tension probe for live imaging which would differentiate the vesicular and PT membrane tension, and use of vesicle targeting pH sensitive fluorescence probe (Phluorins) combined with TIRFM and STICS, which would give some clue on the mode of membrane dynamics at PT–tip.

## Author Contributions

PA designed the manuscript, prepared the figures, and wrote the manuscript. XL carried out the *in vitro* pollen tube and root hairs observations under the guidance of PA. RK assisted during the manuscript preparation and revision. All authors contributed to the article and approved the submitted version.

## Conflict of Interest

The authors declare that the research was conducted in the absence of any commercial or financial relationships that could be construed as a potential conflict of interest.
